# Delayed Radial Nerve Palsy Following a Low-Impact Mechanical Fall Managed With Early Ultrasound-Guided Hydrodissection: A Case Report

**DOI:** 10.7759/cureus.101361

**Published:** 2026-01-12

**Authors:** Stephanie Marrero Borrero, Jose M Mariani, Miguel F Agrait Gonzalez

**Affiliations:** 1 Emergency Medicine, Centro Medico Episcopal San Lucas, Ponce, PRI

**Keywords:** acute radial nerve palsy, blunt trauma, hydrodissection, olecranon bursitis, pain in elbow, radial nerve injury, wrist drop

## Abstract

A 43-year-old male presented to the emergency department due to progressive weakness of the left hand and wrist following blunt trauma to the elbow around three weeks prior to evaluation. He was initially evaluated at the time of injury with radiographs of the elbow and forearm, which were reported to be normal. Due to worsening weakness, he sought care at additional emergency departments and with a neurologist who performed a CT, repeat radiographs, and eventually an MRI of the elbow, all of which revealed no acute findings. At our facility, point-of-care ultrasound evaluation of the elbow demonstrated radial nerve compression just proximal to the joint. Ultrasound-guided hydrodissection of the radial nerve was performed, after which the patient showed improvement in range of motion and strength over the following days. With early initiation of physical therapy, the patient regained full range of motion against gravity within one month and achieved complete recovery within three months. Recognition and prompt management of peripheral nerve compression requires a multidisciplinary approach, involving emergency medicine, physical medicine and rehabilitation, and neurology. Early intervention may help prevent long-term dysfunction and aid in more rapid recovery of compressive neuropathies.

## Introduction

Radial nerve palsy is the most common peripheral neuropathy associated with humeral shaft trauma, whether direct or indirect [1-3]. Radial nerve palsy classically presents with motor impairment, including wrist drop, loss of finger extension, and possible sensory deficits on the dorsum of the hand. These findings frequently result from neurapraxia due to external compression of the radial nerve [4].

Although both radial nerve palsy and olecranon bursitis may develop after trauma, their concurrent presentation following a single low-impact injury is uncommon. Radial nerve palsy usually arises from compression, whereas olecranon bursitis typically follows repetitive microtrauma or a discrete blow to the olecranon itself [5]. Timely intervention is important to optimize functional recovery and prevent long-term disability. Clinical improvement may not be evident for up to six months because nerve regeneration progresses slowly. Complete recovery may take up to one year, and surgical exploration is generally recommended when no clinical improvement occurs after four to six months [1-3]. Management is usually conservative, with surgical intervention reserved for recalcitrant cases. Initial therapy for peripheral nerve compression includes activity modification, splinting, nerve gliding exercises, or physical therapy. Although spontaneous recovery is common, it may take several months, resulting in prolonged functional impairment. Perineural hydrodissection is an emerging therapy allowing mechanical release of nerve adhesions and decompression before surgical options are considered [6-7].

This case highlights the importance of early recognition of peripheral nerve compression. It demonstrates how ultrasound-guided hydrodissection, combined with physical therapy, may facilitate more rapid functional recovery than conservative measures alone.

## Case presentation

**Figure 1:**
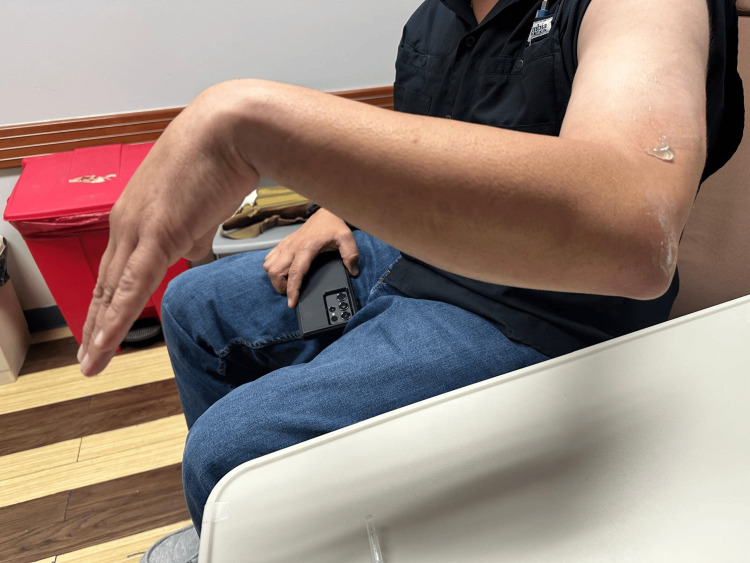
Wrist drop on emergency department evaluation

A right-hand-dominant 43-year-old male businessman with no significant past medical history presented after sustaining a ground-level fall onto his left elbow. He fell with the arm in flexion, resulting in blunt trauma to the elbow. Initially, he reported no motor deficits; however, three days later, he developed progressive loss of strength in wrist extension, eventually developing wrist drop accompanied by marked swelling around the olecranon (Figure 1).

He was first evaluated at an outside emergency department, where plain radiographs of the elbow and a head CT showed no acute abnormalities. The patient was subsequently evaluated by a neurologist, who ordered MRI studies of the elbow and forearm, which showed no structural injury. Due to continued symptoms, he visited a second emergency department, where again radiographs were unremarkable. Three weeks post-injury, he presented to our facility for further evaluation. None of the prior imaging was available at the time of our evaluation.

On arrival, vital signs were stable. Examination of the left upper extremity revealed visible swelling over the elbow and olecranon process. The patient had a normal range of motion of the elbow, though flexion elicited discomfort. No overlying skin break, warmth, or purulent discharge was noted. Neurologic examination demonstrated loss of active wrist extension, while sensation remained intact. Capillary refill was normal. Examination of the right upper extremity and lower limbs was unremarkable.

As prior imaging was not available, the patient underwent plain radiographs, which showed no fracture or dislocation. Point-of-care ultrasound of the elbow demonstrated a fluid collection over the olecranon, consistent with olecranon bursitis, along with the physical exam findings of radial nerve palsy.

**Figure 2:**
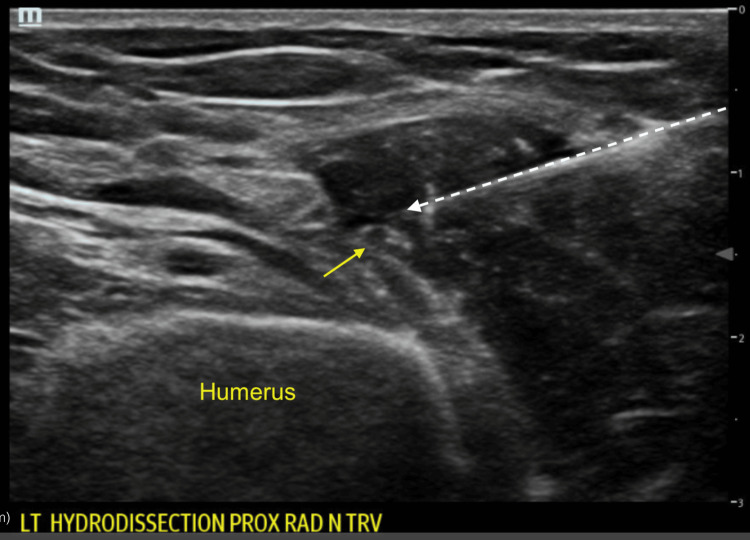
Transverse ultrasound view of early radial nerve hydrodissection showing the radial nerve (yellow arrow) and needle (white dotted arrow)

Given the suspicion of acute compressive neuropathy, ultrasound was used to evaluate the nerve itself, with an area of focal compression visualized. Ultrasound-guided hydrodissection of the radial nerve was then performed in the emergency department (Figure 2).

**Figure 3:**
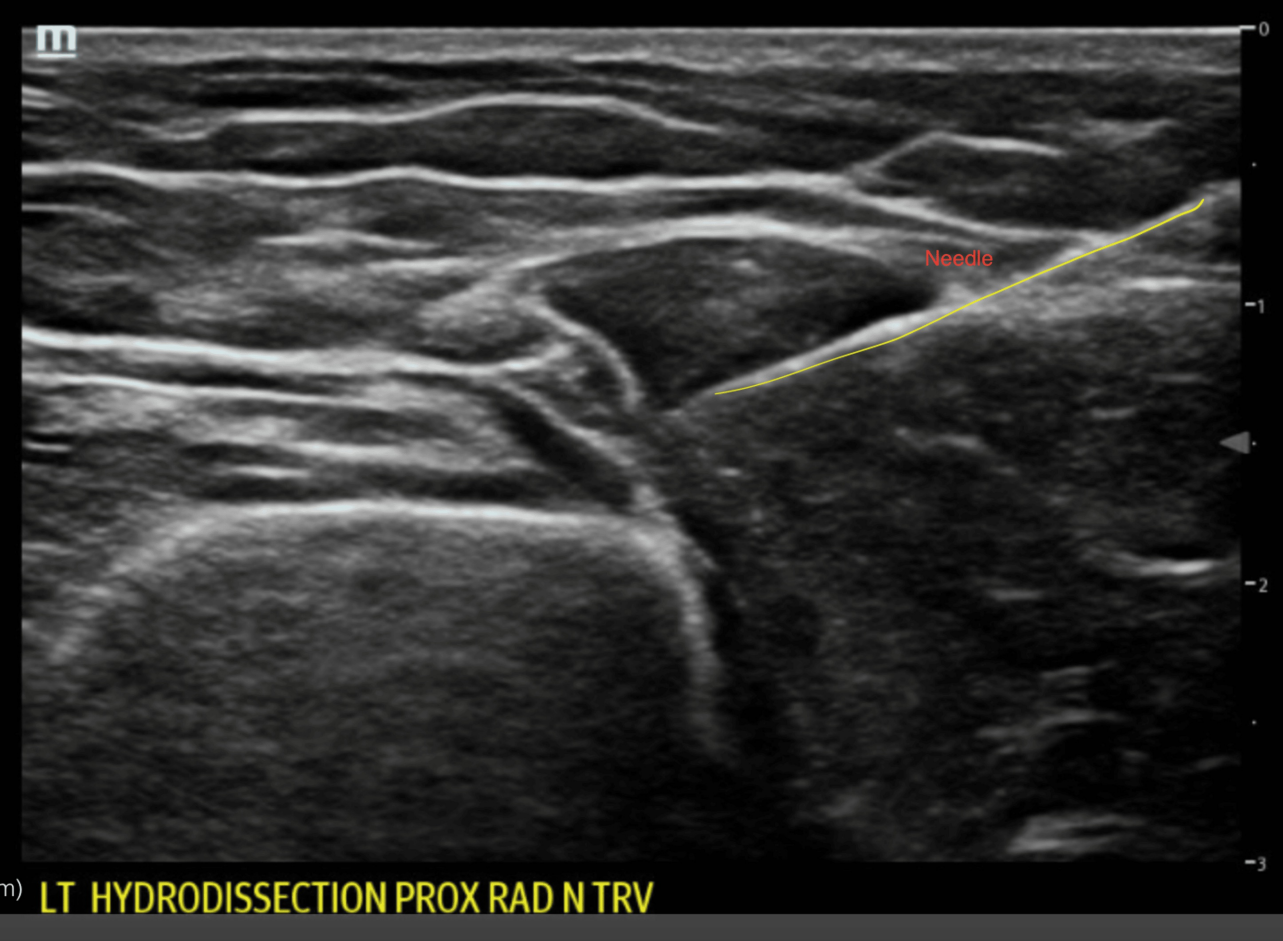
Ultrasound showing hydrodissection with additional fluid adjacent to the radial nerve

A 25-gauge needle was advanced to the area of the radial nerve. The following injectate was administered: 8 mL of 5% dextrose (D5W), 2 mL of 1% lidocaine (without epinephrine), and 1 mL of dexamethasone (4 mg/mL). Hydrodissection achieved successful separation of the radial nerve from the surrounding fascia and edematous tissue (Figures 2-3). Aspiration of the olecranon bursa yielded 8 mL of clear serous fluid, negative for crystals or infection. A wrist splint was provided, and the patient was referred to outpatient physical therapy.

Within 24 hours of the hydrodissection procedure, the patient reported significant partial improvement in wrist extension and a reduction in swelling over the olecranon bursa. He was discharged with a wrist splint to prevent contractures while maintaining metacarpophalangeal joint mobility. At the six-week follow-up visit, the patient demonstrated continued improvement in motor function, with wrist extension graded as Medical Research Council 3/5. He continued physical therapy, focused on strengthening the affected extremity, and used the wrist splint during work activities. At the three-month follow-up, the patient reported complete resolution of symptoms and has returned to full-time employment without any limitations. He states he completed eight sessions of physical therapy following the hydrodissection.

## Discussion

Ultrasound-guided hydrodissection has gained attention as a minimally invasive and potentially effective alternative in selected neuropathies. Hydrodissection involves the injection of fluid, typically normal saline, D5W, or injectable mixtures including anesthetic or steroid, to mechanically separate the nerve from surrounding structures, restore gliding, and reduce compression. Utilizing POCUS, the technique provides real-time visualization, targeted decompression, and potential analgesic benefit when D5W is used. In addition to the potential benefits of mechanical separation, D5W modulates neurogenic inflammation and nociceptive signaling and may outperform saline or corticosteroid injections in certain compressive neuropathies [6-9].

This case is noteworthy because it illustrates delayed radial nerve palsy after blunt elbow trauma in association with olecranon bursitis and demonstrates the potential role of ultrasound-guided hydrodissection as an early decompressive strategy. Most closed radial nerve injuries are managed expectantly, as the majority of patients recover spontaneously over several months, reflecting neurapraxia or low-grade axonotmesis rather than nerve transection [3,10-12]. However, prolonged observation often entails substantial functional impairment, especially in individuals whose work depends on upper-extremity strength and dexterity, as in the present patient.

In our case, the mechanism appears to be acute compressive neuropathy related to post-traumatic swelling and olecranon bursitis rather than direct laceration or high-energy open injury. The patient initially had no motor deficit but developed progressive wrist drop over several days, suggesting an ongoing subacute process rather than a direct nerve injury. The prompt partial improvement in wrist extension within 24 hours of hydrodissection, followed by ongoing gains with splinting and physical therapy, supports the hypothesis that mechanical decompression of the nerve contributed meaningfully to recovery.

Evidence for hydrodissection has grown most robustly in entrapment neuropathies such as carpal tunnel syndrome (CTS). Lam et al. systematically reviewed ultrasound-guided interventions for CTS and found that perineural injections, including hydrodissection, led to significant improvements in pain and function, with favorable safety profiles [8]. Chao et al. reported that hydrodissection with D5W for persistent or recurrent CTS produced durable symptom relief and functional improvement in many patients, suggesting that D5W may offer advantages over saline or steroid in some settings [9]. More recently, broader reviews of ultrasound-guided perineural hydrodissection across multiple peripheral nerves have reinforced its potential to mechanically release adhesions, reduce neuropathic pain, and improve nerve mobility, with a low incidence of serious complications [6-7,11].

Radial nerve-specific data are more limited but encouraging. Su et al. described the use of shear wave elastography to guide perineural hydrodissection in two cases of radial nerve entrapment, demonstrating both technical feasibility and clinical improvement [6]. Gill et al. presented a case series and scoping review of patients with radial tunnel syndrome treated with ultrasound-guided hydrodissection of the radial nerve, noting complete resolution of symptoms without the need for surgical decompression [7]. These reports, although small and largely observational, suggest that hydrodissection may have particular utility in radial nerve pathology, where the nerve is constrained within a tight fibro-osseous space or by peri-neural soft-tissue thickening.

Our case extends this literature by applying ultrasound-guided radial nerve hydrodissection in the acute post-traumatic setting within the emergency department. Traditionally, emergency physicians have focused on diagnosing fractures and dislocations and initiating splinting, analgesia, and referral for suspected nerve injuries. The availability of high-resolution point-of-care ultrasound (POCUS) now allows real-time visualization not only of bony structures and effusions but also of peripheral nerves [10]. In this patient, POCUS was used to identify both the olecranon bursitis and the radial nerve at the site of compression, enabling a targeted procedure during the index ED visit. This approach potentially shortens the time to decompression compared with delayed referral to radiology or outpatient specialty clinics.

Despite these promising features, several limitations must be acknowledged. First, the natural history of closed radial nerve palsy, particularly in lower-energy injuries, often includes spontaneous recovery, making it difficult to determine how much of this patient’s improvement was attributable to hydrodissection rather than to the passage of time and rehabilitation [1,9-12]. Second, although ultrasound strongly supported the diagnosis of a compressive neuropathy and guided therapy, electrodiagnostic studies remain essential for characterizing lesion severity, prognostication, and ruling out more proximal causes such as cervical radiculopathy or posterior cord lesions. Third, high-quality comparative data for hydrodissection are sparse; most reports to date are case reports, small case series, or observational cohorts without control groups.

From a practical standpoint, this case underscores several considerations for emergency and sports medicine clinicians. Peripheral nerve evaluation should be incorporated into the sonographic assessment of the traumatized extremity when there is unexplained weakness, sensory deficit, or disproportionate pain. When a focal compressive etiology, such as bursitis, hematoma, or thickened fascia, is identified, early multidisciplinary discussion with physical medicine and rehabilitation, neurology, and orthopedics or hand surgery can help determine whether hydrodissection is appropriate.

Future research should aim to define the role of ultrasound-guided hydrodissection in acute traumatic neuropathies more precisely. Priorities include prospective studies comparing hydrodissection plus standard care versus standard care alone, standardized protocols for injectate composition and volume by nerve and anatomical region, and longer-term follow-up using both clinical and electrodiagnostic outcomes. Our case supports its feasibility and potential benefit in the acute setting and highlights the expanding therapeutic capabilities of POCUS within the emergency department.

## Conclusions

Radial nerve palsy may present in a delayed fashion after blunt elbow trauma and can occur in association with olecranon bursitis, a rare clinical scenario. Conservative management remains the standard for closed-injury radial nerve palsy, but recovery can take months, often with significant functional impairment during this period. Ultrasound-guided hydrodissection offers a minimally invasive, real-time approach to peripheral nerve decompression, with growing evidence supporting its safety and efficacy. Early application of hydrodissection in the emergency department or clinic setting may accelerate recovery, reduce disability, and provide an alternative to prolonged observation or invasive surgery in selected cases.
